# Viral nucleic acid analysis with PCR in lacrimal tissue and nasal swab samples of primary acquired nasolacrimal duct obstruction cases

**DOI:** 10.1177/1120672119882331

**Published:** 2019-10-21

**Authors:** Özge Yanık, Banu Hoşal, Alper Tekeli, Hilal Nalcı

**Affiliations:** 1 Department of Ophthalmology, Ankara University School of Medicine, Ankara, Turkey; 2 Department of Medical Microbiology, Ankara University School of Medicine, Ankara, Turkey; 3 Ürgüp State Hospital, Nevşehir, Turkey

**Keywords:** Adenovirus, coronavirus, polymerase chain reaction, primary acquired nasolacrimal duct obstruction, respiratory syncytial virus

## Abstract

**Purpose:**

To evaluate the role of viral infections in the pathogenesis of primary acquired nasolacrimal duct obstruction.

**Methods:**

The study included 48 patients diagnosed with primary acquired nasolacrimal duct obstruction undergoing dacryocystorhinostomy surgery. Prior to dacryocystorhinostomy surgery, nasal swab sample was taken from the inferior meatus at the same side. During dacryocystorhinostomy, tissue biopsy sample (2 × 2 mm) was taken from the junction area of the lacrimal sac and nasolacrimal duct. Following nucleic acid extraction, polymerase chain reaction was performed.

**Results:**

The patients consisted of 9 (18.8%) men and 39 (81.2%) women with a mean age of 51.0 ± 14.3 years. Qualitative polymerase chain reaction showed viral genome in the nasal swabs of 10 (20.8%) patients, including coronavirus 229E (three cases), coronavirus HKU1 (two cases), respiratory syncytial virus (two cases), coronavirus OC43 (one case), coronavirus NL63 (one case), and adenovirus (one case). In the dacryocystorhinostomy samples, viral genomes were detected in four (8.3%) cases, including respiratory syncytial virus (two cases), coronavirus HKU1 (one case), and adenovirus (one case). There was a statistically significant agreement between nasal mucosal swab and dacryocystorhinostomy biopsy samples in terms of respiratory syncytial virus positivity (kappa = 1.000, *p* = 0.001).

**Conclusion:**

Although the viral genome was detected in the samples, a direct relationship between viruses and pathogenesis of primary acquired nasolacrimal duct obstruction could not be revealed because of the low number of positive results. However, considering the profibrotic characteristics of specific viruses such as respiratory syncytial virus and adenovirus, viral infections may be one of the many predisposing factors of primary acquired nasolacrimal duct obstruction.

## Introduction

Primary acquired nasolacrimal duct obstruction (PANDO) is the most common cause of epiphora in adults.^1^ A variety of anatomical, endocrine, environmental, and socioeconomic risk factors have been suggested in the etiology; however, the precise pathogenesis of PANDO has not yet been elucidated. Previous histopathological studies have revealed chronic inflammation of the nasolacrimal duct and the development of secondary fibrosis on the ground of this inflammation.^2–4^

PANDO has been reported to be able to occur secondary to unrecognized low-grade dacryocystitis.^5^ In one experimental model of dacryocystitis, the number of collagen fibers and fibrocytes in the lamina propria had increased 3 months after a *Staphylococcus aureus* inoculation.^6^ Viruses, as superior to bacteria, have capability to extend beyond the epithelium to involve the elastic tissue of the lamina propria, causing inflammation and cicatricial changes in the mucosa.^5^

Recently, viral infections have been reported as the possible trigger factors in the pathogenesis of several diseases, such as idiopathic pulmonary fibrosis, dilated cardiomyopathy, and arrhythmogenic right ventricular dysplasia, characterized by inflammation and secondary fibrosis in their histopathological examinations.^7–10^ A similar mechanism may be possible for the pathogenesis of PANDO which has common histopathological characteristics with these diseases.

The respiratory mucosa and nasolacrimal duct epithelium has similar immunological and histological features. First, the nasolacrimal duct is a direct extension of the nasal mucosa and has been shown to include ectopic nasal epithelial cells.^11^ In addition, respiratory mucosa and nasolacrimal duct has the same sialic acid sequences in the end of the glycoproteins that serve almost as a receptor for infectious agents.^12^ These close anatomical and immunological relationships may cause an expansion of the virus inoculation to the nasolacrimal duct during upper respiratory tract infections. Similarly, viral conjunctivitis may easily extend into the lacrimal sac and nasolacrimal duct mucosa in the direction of tear drainage. A higher frequency of histories of infectious (viral or bacterial) conjunctivitis in cases with PANDO was previously reported.^13^ The aim of the present study was to evaluate the role of respiratory and ocular viruses in the PANDO pathogenesis.

## Methods

This prospective study included 48 patients diagnosed with symptomatic PANDO who underwent dacryocystorhinostomy (DCR) surgeries. Approval from the ethics committee was obtained, and the study was performed in adherence to the tenets of the Declaration of Helsinki. Informed consent was obtained from each of the participants prior to enrollment.

### Patient selection

All the patients underwent complete ocular and periocular examinations. The PANDO diagnosis was confirmed via lacrimal system probing and irrigation. All factors that could cause the secondary acquired nasolacrimal duct obstruction were questioned and evaluated in detail. Also, all patients underwent otorhinolaryngology consultation to rule out intranasal mass lesions. Any cases with suspected or positive history and/or findings in favor of other causes of nasolacrimal duct obstruction and cases with intranasal mass lesions were excluded. Those patients with histories of viral conjunctivitis or upper respiratory tract infections within the previous 3 months were excluded. In addition, those patients with ocular diseases that could have affected the lacrimal drainage system were also excluded.

### Sample collection

Prior to the DCR surgery, a nasal mucosal swab sample was taken from the same side of the inferior meatus of each patient. During the DCR surgery, tissue biopsy samples (2 × 2 mm) were taken from the junction area of the lacrimal sac and the proximal nasolacrimal duct. The samples were immediately transferred to the Department of Medical Microbiology under cold chain.

### Nucleic acid extraction

The viral genome extraction was performed with an EZ1 Virus Mini Kit v2.0 (Qiagen, Hilden, Germany) using an EZ1 Advanced XL Robotic Workstation (Qiagen) according to the manufacturer’s instructions. After performing the extraction, the samples were frozen at −80°C.

### Polymerase chain reaction

Polymerase chain reaction (PCR) using real-time technology for qualitative analysis of the pathogens was performed with 5-tube multiplex PCR kit “FTD Respiratory 21 (Fast-track diagnostics Ltd, Luxembourg)” and 2-tube multiplex PCR kit “FTD Eye” on the “Rotor-Gene^®^ Q (Qiagen, Hilden, Germany)” device in accordance with the manufacturer’s recommendations. FTD Respiratory 21 kit includes influenza A virus, influenza B virus, influenza A (H1N1) virus, human rhinovirus, human coronavirus NL63, human coronavirus 229E, human coronavirus OC43, human coronavirus HKU1, human parainfluenza 1, human parainfluenza 2, human parainfluenza 3, human parainfluenza 4, human metapneumoviruses A/B, human bocavirus, *Mycoplasma pneumoniae*, human respiratory syncytial viruses (RSVs) A/B, human adenovirus, enterovirus, human parechovirus, and internal control. FTD Eye kit includes herpes simplex virus 1, herpes simplex virus 2, varicella zoster virus, *Chlamydia trachomatis*, human adenovirus, and internal control. Reactions which were in the desired cycle threshold and had exponential phase were regarded as positive.

### Statistical analysis

The statistical analysis was performed using SPSS software for Windows version 15.0 (SPSS, Inc, Chicago, IL). Descriptive statistics were expressed as mean ± standard deviation or median (minimum–maximum) for continuous variables and as the number of observations and percentage for categorical variables. Differences in categorical variables were assessed by Pearson’s chi-square test or Fisher’s exact test. Statistical agreement between virus positivity in DCR biopsy specimens and nasal swab specimens was tested by the kappa coefficient (κ). A value of *p* < 0.05 was considered statistically significant.

## Results

The study subjects consisted of 9 (18.8%) males and 39 (81.2%) females with a mean age of 51.0 ± 14.3 years. The female-to-male ratio was 4.3. The mean age of the females was 48.0 ± 13.4 years, and the mean age of the males was 63.7 ± 11.2 years. The mean age difference between the two genders was statistically significant (*p* = 0.002). The patients’ demographic data are summarized in Table 1. Most of the females were in the 40- to 49-year-old age group (13 cases, 33.3%), and most of the males were in the 60 years or older age group (7 cases, 77.8%). A statistically significant difference was found between the age distributions of the patients based on the sex (*p* = 0.008).

**Table 1.** Demographical characteristics of the cases.

	Female, *n* = 39	Male, *n* = 9	*p* value
Age			**0.002**
Mean ± SD	48.0 ± 13.4	63.7 ± 11.2	
Median	48	65	
Range	21–75	45–81	
Age group (years)			**0.008**
20–39	10 (25.6%)	0 (0.0%)	
40–49	13 (33.3%)	1 (11.1%)	
50–59	9 (23.1%)	1 (11.1%)	
⩾60	7 (17.9%)	7 (77.8%)	
Laterality			1.000
Unilateral	25 (64.1%)	6 (66.7%)	
Bilateral	14 (35.9%)	3 (33.3%)	

SD: standard deviation.

Bold values denote statistical significance at the *p* < 0.05 level.

The evaluation of the symptoms and clinical findings of the PANDO cases showed that bilateral PANDOs were present in 17 (35.4%) of the cases. With regard to the unilateral cases, 18 (37.5%) patients had right-sided involvement and 13 (27.1%) had left-sided involvement. Epiphora was the most frequent clinical finding that was seen in all the cases. The other examination findings related to the lacrimal drainage system obstruction were punctual regurgitation with sac compression in 31 cases (64.6%) and localized swelling in 13 cases (27.1%).

The PCR results showed the presence of viral genome in the nasal swab samples of 10 (20.8%) of the patients. The viruses that were detected included human coronavirus 229E in three cases, human coronavirus HKU1 in two cases, human RSV in two cases, human coronavirus OC43 in one case, human coronavirus NL63 in one case, and a human adenovirus in one case. In the DCR biopsy samples, viral genomes were isolated in four (8.3%) of the cases, including human RSV in two cases, human coronavirus HKU1 in one case, and a human adenovirus in one case. The demographic characteristics of the cases in which viral genome was detected and the side with the viral genome are summarized in Table 2. In terms of the viral genome positivity, there was no statistically significant agreement between the nasal mucosal swab and DCR biopsy samples (*p* = 0.187) (Table 3). In addition, there were no statistically significant differences between the males and females in terms of the viral genome positivity in the nasal mucosal swab or DCR biopsy samples (*p* = 0.370 and *p* = 1.000, respectively). The RSV was discovered in both the nasal mucosa and DCR biopsy samples in two patients (4.2%), and there was statistically significant agreement between the nasal mucosa and DCR biopsy samples in terms of the RSV positivity (kappa = 1.000, *p* = 0.001). In the other two patients in whom a virus (human coronavirus HKU1 in one and a human adenovirus in the other) was detected in the DCR biopsy sample, the virus was not detected in the nasal mucosa.

**Table 2.** The demographic characteristics of the viral genome–detected cases and the localization of the viral genome presence.

Patient	Age (years)	Gender	Detected virus	
			Nasal mucosal swab	DCR biopsy specimen
1.	48	F	–	Adenovirus
2.	72	F	–	Coronavirus HKU1
3.	50	F	RSV A/B	RSV A/B
4.	34	F	RSV A/B	RSV A/B
5.	48	F	Coronavirus HKU1	–
6.	62	F	Coronavirus HKU1	–
7.	81	M	Coronavirus 229E	–
8.	44	M	Coronavirus 229E	–
9.	48	F	Coronavirus 229E	–
10.	71	M	Coronavirus OC43	–
11.	41	F	Coronavirus NL63	–
12.	71	F	Adenovirus	–

DCR: dacryocystorhinostomy; RSV: respiratory syncytial virus.

**Table 3.** Comparison of nasal mucosal swab and dacryocystorhinostomy biopsy specimens regarding viral genome presence.

DCR specimen samples	Nasal mucosal swab samples		Total
	Viral genome absent	Viral genome present	
Viral genome absent	36 (75.0%)	8 (16.7%)	44 (91.7%)
Viral genome present	2 (4.2%)	2 (4.2%)	4 (8.3%)
Total	38 (79.2%)	10 (20.8%)	48 (100.0%)
*p* value			0.187

DCR: dacryocystorhinostomy.

## Discussion

PANDO is the most common cause of acquired nasolacrimal duct obstruction in the adult.^3^ The exact mechanisms leading to chronic inflammation and secondary fibrosis, which are the predominant findings of the PANDO histopathology,^2–4^ have not yet been elucidated. The only previous study that evaluated the viral presence in PANDO was performed by Kashkouli et al.^14^ They examined the biopsy specimens of 51 PANDO cases for the presence of herpes simplex virus 1, herpes simplex virus 2, and human papillomavirus. However, the immunohistochemistry results were negative for the viruses in all the cases. To the best of our knowledge, the present study was the first to report the presence of respiratory viral genomes directly in the proximal nasolacrimal duct mucosa of four patients with PANDO.

Paulsen et al.^11^ speculated that ascending infection of ectopic nasal epithelial cells in the nasolacrimal duct mucosa during nasal inflammation could be the starting point of dacryostenosis. Then, this inflammation may initiate dysfunction of the cavernous body with reactive hyperemia, edema of the mucous membrane, and temporary occlusion of the lacrimal passage. Repeated episodes of dacryocystitis may end up with the structural epithelial and subepithelial changes. Considering the fact that acute viral respiratory tract infections are the most common illnesses in all individuals, repetitive inflammation caused by the nasal infection may be direct or indirect stimuli for the nasolacrimal duct obstruction, especially in predisposed individuals.

In the present study, two of the patients exhibited the RSV genome in both the nasal mucosal swab and the DCR biopsy specimens. RSV is a segmental, negatively polarized RNA virus.^15^ It has the capability of affecting the cytokine production profile of adaptive immune system cells by shifting the T helper (Th) 1 / Th 2 balance toward Th 2 cytokines and immunoglobulin E.^16^ It has also been shown to alter the biological activity of macrophages.^17^ Furthermore, matrix metalloproteinase 9 (MMP-9), a profibrotic enzyme has been shown to increase in the epithelium after an RSV infection.^18^ When all these mechanisms are considered together, RSV has the capability to trigger inflammation and fibrosis development by both shifting the Th balance toward the production of the profibrotic cytokines, interleukin (IL)-4 and IL-13, and increasing the synthesis of connective tissue enzymes, such as MMP-9.^18,19^

Adenovirus DNA was detected in the DCR biopsy specimen from one case and in the nasal swab sample from one case. Adenoviruses, which are common respiratory tract viruses with more than 50 serotypes, have been proven to cause latent infections, especially in lymphoid tissues.^20–22^ Early region 1A, which is a viral gene product of adenoviruses, has been reported to promote the production of profibrotic mediators, such as connective tissue growth factor and transforming growth factor-β mRNA on the bronchial epithelium.^23^ In the case of a latent adenovirus infection, the continuous release of these profibrotic mediators may trigger fibrosis development. In addition, the nasolacrimal duct is surrounded by a mucosa-associated lymphoid tissue, which is believed to provide immunomodulation, and damage to this structure has been associated with the development of PANDO.^24,25^ Because adenoviruses have been proven to cause latent infections in lymphoid tissues,^22^ a persistent viral infection in this special lymphoid tissue may lead to a chronic inflammatory process resulting in injury and scar formation.

One case exhibited human coronavirus HKU1 RNA in the DCR biopsy sample. In this case, no virus was detected in the nasal swab. When considering the fact that the ocular mucosa is one of the coronavirus transmission routes,^26^ the human coronavirus HKU1 detected in the DCR biopsy material may be a case of an asymptomatic infection through the ocular surface. In our study, the seven patients (14.6%) with coronaviruses in their respiratory tracts were believed to be asymptomatically infected cases.

This was a pilot study, and the major study limitations were the small sample size and the low number of viral positives in the DCR samples, which prevented us from establishing a definitive association between PANDO and viral infections. Sexual differences in cytokine responses to infection may also have a role in determining susceptibility to viruses.^27^ Therefore, further studies including large number of cases with equal gender and age distribution and usage of quantitative real-time PCR analysis for suspected viruses to decide the clinical stage of the infection are needed. Animal models in which virus inoculation is performed directly into the lacrimal sac may also help to assess the mucosal changes that viruses can reveal in this region. Moreover, population-based studies with a long follow-up time that evaluate the time period prior to the disease development may help to elucidate the precise pathogenesis of PANDO.

In conclusion, the predominant findings in the PANDO histopathology are inflammation and secondary fibrosis, but the factors that may trigger the critical process have not yet been elucidated. Many inflammatory, profibrotic, and immunological pathways may have roles in the pathogenesis of PANDO. In the present study, although the viral genome was detected in the DCR samples, due to the low number of positive results, a direct relationship between the presence of viruses and the PANDO pathogenesis could not be established. However, considering the profibrotic characteristics of specific viruses such as RSV and adenovirus, viral infections may be one of the many predisposing factors of PANDO.
